# Using nontargeted LC-MS metabolomics to identify the Association of Biomarkers in pig feces with feed efficiency 

**DOI:** 10.1186/s40813-021-00219-w

**Published:** 2021-06-02

**Authors:** Jie Wu, Yong Ye, Jianping Quan, Rongrong Ding, Xingwang Wang, Zhanwei Zhuang, Shenping Zhou, Qian Geng, Cineng Xu, Linjun Hong, Zheng Xu, Enqin Zheng, Gengyuan Cai, Zhenfang Wu, Jie Yang

**Affiliations:** 1 College of Animal Science and National Engineering Research Center for Breeding Swine Industry, South China Agricultural University, Guangzhou, 510642 China; 2 Guangdong Provincial Laboratory of Lingnan Modern Agricultural Science and Technology, Guangzhou, 510642 China; 3 State Key Laboratory for Conservation and Utilization of Subtropical Agro-bioresources, Guangzhou, 510642l China; 4 Guangdong Provincial Key Laboratory of Agro-animal Genomics and Molecular Breeding, Guangzhou, 510642 China

**Keywords:** Feed efficiency, Pig, LC-MS, WGCNA

## Abstract

**Background:**

Improving feed efficiency is economically and environmentally beneficial in the pig industry. A deeper understanding of feed efficiency is essential on many levels for its highly complex nature. The aim of this project is to explore the relationship between fecal metabolites and feed efficiency-related traits, thereby identifying metabolites that may assist in the screening of the feed efficiency of pigs.

**Results:**

We performed fecal metabolomics analysis on 50 individuals selected from 225 Duroc x (Landrace x Yorkshire) (DLY) commercial pigs, 25 with an extremely high feed efficiency and 25 with an extremely low feed efficiency. A total of 6749 and 5644 m/z features were detected in positive and negative ionization modes by liquid chromatography-mass spectrometry (LC/MS). Regrettably, the PCA could not classify the the samples accurately. To improve the classification, OPLS-DA was introduced. However, the predictive ability of the OPLS-DA model did not perform well. Then, through weighted coexpression network analysis (WGCNA), we found that one module in each positive and negative mode was related to residual feed intake (RFI), and six and three metabolites were further identified. The nine metabolites were found to be involved in multiple metabolic pathways, including lipid metabolism (primary bile acid synthesis, linoleic acid metabolism), vitamin D, glucose metabolism, and others. Then, Lasso regression analysis was used to evaluate the importance of nine metabolites obtained by the annotation process.

**Conclusions:**

Altogether, this study provides new insights for the subsequent evaluation of commercial pig feed efficiency through small molecule metabolites, but also provide a reference for the development of new feed additives.

**Supplementary Information:**

The online version contains supplementary material available at 10.1186/s40813-021-00219-w.

## Background

Feed remains the main input cost in all animal production. Additionally, pork is one of the most important sources of meat for human beings. To cope with increasing market demand and breeding costs, improving the feed efficiency (FE) of pigs has always been a concern of breeders. Since FE cannot be measured directly for its complex pleiotropy, the feed conversion ratio (FCR) and residual feed intake (RFI) are often used as alternative selection conditions for FE [1–4]. However, although RFI traits have a strong genetic correlation with FCR (0.76 to 0.99) [5], a unified indicator for evaluating feed efficiency has not yet been determined.

At present, there are various omics methods to explore the molecular mechanisms that affect the feed efficiency of pigs, including transcriptomics [6, 7], genomics [5] and 16S rRNA gene sequencing [8]. However, metabolomics is rarely used to study pig feed efficiency phenotypes. Metabolites produced by the intestinal microbiota are increasingly recognized as an important part of human physiology [9]. As the downstream of the gene regulation network and protein interaction network, the metabolite can provide more detailed biological terminal information. By analyzing the changes in the expression of metabolites, it can help researchers find novel biomarkers and further understand the currently known metabolic pathways so that they can be applied to the study of various apparent traits. Furthermore, fecal metabolites are the final products of the metabolism of cells and intestinal microbiota, which can help to reflect the absorption and digestion of nutrients by the intestinal flora and digestive tract more comprehensively. Finally, the analysis of fecal metabolomics provides a noninvasive way to study the correlation between biological traits and metabolites. Therefore, the fecal metabolome can not only partially explain the composition of the gut microbiota but also be used as biomarkers to investigate the relationship between gut microbial metabolism and host phenotypes [10].

Duroc × (Landrace × Yorkshire) (DLY) commercial pigs has the advantages of high lean meat rate, low backfat thickness, high water holding capacity (WHC), suitable pH value (pH 1 > 5.9; 6.2 > pH 2 > 5.5) and IMF content (about 2.5%) for pork, etc. [11]. Desired carcass and meat quality traits makes DLY pigs currently account for the largest sales share in the Chinese pork market. Therefore, exploring the factors that affect the feed efficiency of DLY pigs has great significance for improving the economic benefits of the swine industry.

Liquid chromatography-mass spectrometry (LC/MS) is a relatively high-resolution separation analysis technique. Because of its high sensitivity, wide dynamic range, and lack of derivatization, LC-MS analysis has become a common technique for metabolomics research [12, 13]. However, few studies have used LC/MS technology to analyze the relationship between the fecal metabolome and feed efficiency. Hence, in this project, we analyzed the metabolomics of feces based on high and low feed efficiency groupings, aiming to unearth some small molecular metabolites that can evaluate feed efficiency and provide a new perspective for feed management in the pig industry.

## Results

### Difference analysis of FE-related traits

**Fig. 1:**
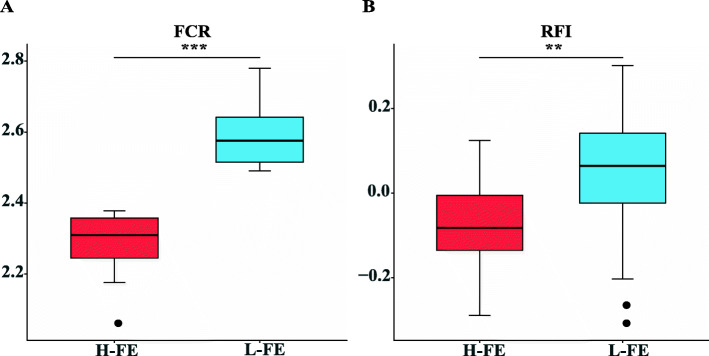
Boxplot of feed efficiency (FE)-related phenotypes. Differences in (**A**) FCR and (**B**) RFI trait between high- and low-FE groups. H-FE, high-feed efficiency; L-FE, low-feed efficiency; FCR, feed conversion ratio; RFI, residual feed intake. *** *p* < 0.0001. **p < 0.01

The summary of FE-related phenotypic data, such as average daily feed intake (ADFI), body weight (BW) and average daily gain (ADG), are shown in Table 1 and Supplementary Table S1. The calculated FCR and RFI data were used to compare the differences between the high- and low-FE groups. There were significant differences in the FCR phenotype between these two groups (*p* < 0.0001, ANOVA) (Fig. 1A). Similarly, the RFI of the low-FE group was significantly higher than that of the high-FE group (*p* = 0.0019, unpaired t-test) (Fig. 1B).

**Table 1:** Phenotypic data for the two groups of female pigs selected for high (*n* = 25) or low feed efficiency (*n* = 25)

Class	Variable	Starting day of grow-finishing phase	Final day of grow-finishing phase	Starting BW, kg	Final BW, kg	ADFI, kg/d	ADG, kg/d	RFI	FCR
**High FE**	Average	30.2	111	30.8	100	1.96	0.864	−0.077	2.29
	Standard deviation	4.54	8.26	1.22	0.930	0.167	0.061	0.095	0.079
**Low FE**	Average	29.6	118	30.9	99.7	2.01	0.82	0.038	2.60
	Standard deviation	3.30	8.61	1.38	1.23	0.162	0.098	0.146	0.089
*P*-value	–	0.884	5.59 × 10^−2^	0.984	0.023	0.28	6.43 × 10^−1^	1.90 × 10^−2^	< 0.0001

Statistical significance was determined using one-way ANOVA, unpaired Student t-test and Wilcoxon rank sum test. *BW* body weight; *ADFI* average daily feed intake; *ADG* average daily gain; *RFI* residual feed intake; *FCR*, feed conversion ratio

### Statistics and analysis of different metabolites

**Fig. 2:**
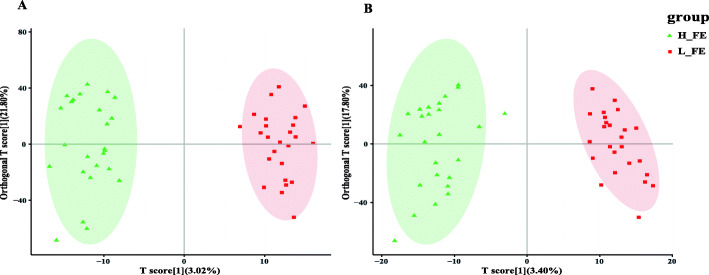
(Orthogonal) Partial Least Squares Discrimination Analysis ((O) PLS-DA) score plots. The analysis was based on LC/MS data of fecal samples from H-FE (green) and L-FE (red) of (**A**) positive and (**B**) negative modes

By liquid chromatography-mass spectrometry, a total of 5644 and 6749 m/z features were detected in negative and positive ionization modes, respectively. Our results showed that there was no clear separation between the high-FE and low-FE groups by PCA analysis (Fig. S1). Furthermore, we used the OPLS-DA model, which is a powerful statistical modeling tool, to identify differential metabolites between the two groups (Fig. 2). The ellipses in score plots for OPLS-DA were defined as the 95% critical limit of the Hotelling T2. For OPLS-DA analysis in both positive and negative ionization modes, the results showed that there was a complete separation between the high-FE group and the low-FE group (R^2^X = 0.357, R^2^Y = 0.966 and Q^2^Y = 0.292 in positive mode; R^2^X = 0.330, R^2^Y = 0.948 and Q^2^Y = 0.178 in negative mode) (Fig. 2A, B). In positive and negative ion modes, the first two components of the OPLS-DA explained 24.8 and 21.2% of the variance, respectively. From the results of OPLS-DA, it can be found that although there was a clear differentiation between the two groups in the positive and negative modes, a lower Q^2^Y value indicated poor predictability and low quality of the model.

### Metabolism network analysis

**Fig. 3:**
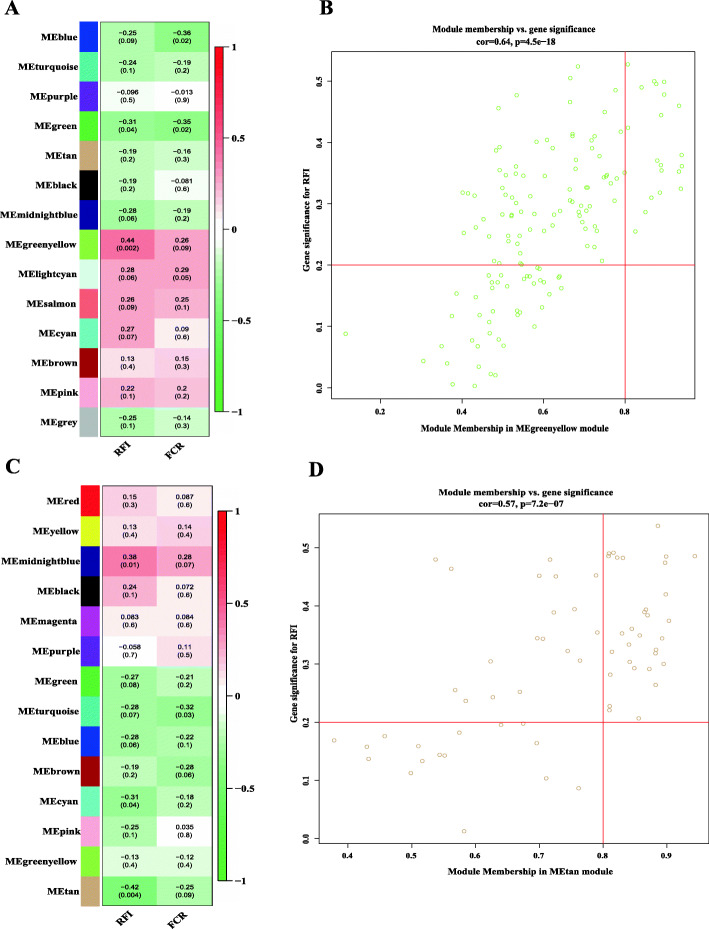
Coexpression network analysis of metabolic features. The left panel of the figure shows the correlation between the module and RFI or FCR in (**A**) negative and (**C**) positive models. The right panel of the figure shows the scatter plot of module membership and the gene significance in (**B**) MEgreenyellow or (**D**) MEtan module. Each row corresponds to ME, and each column corresponds to traits; the number in each module represents the Pearson correlation between the module and RFI or FCR; the number in parentheses represents the *p-*value of the correlation

Referring to the results of the above difference analysis, we tried to use another approach to explore the association between metabolome networks and phenotypes. WGCNA is a method of grouping genes with similar gene expression patterns into a module and finding the hub gene in the module. This analysis was then performed in the positive and negative ion modes. By calculating the adjacency between metabolic features and merging the closer modules, a total of 14 (14) modules were obtained in positive (negative) modes (Fig. S2). Then, by constructing the correlation matrix between the modules and the phenotypic data related to feed efficiency, the module with the closest correlation to the sample traits was identified. Our results showed that the metabolites of the MEtan module had the strongest correlation with RFI traits in positive modes (*r* = 0.42, *p* = 0.004) (Fig. 3A). Similarly, the MEgreenyellow module in negative mode was positively correlated with RFI (*r* = 0.44, *p* = 0.002) (Fig. 3C). In the MEtan (MEgreenyellow) module, a total of 31 (20) metabolic features with important contributions were screened from 65 (145) features (Module Membership = 0.8, Gene significance = 0.2) (Fig. 3B, D). After annotation, we obtained six and three metabolites in the positive and negative modes, respectively (Table S2). The pathways involved in the nine metabolites can be roughly divided into four categories, including lipid metabolism (primary bile acid synthesis, linoleic acid metabolism), vitamin D, glucose metabolism, and others. Separately, three metabolites, 3a,7a,12a-trihydroxy-5b-cholestan-26-al (THC26), 3alpha,7alpha- dihydroxycoprostanic acid (DHCA) and 5β-cholestane-3α,7α,12α,22-tetrol (22-OH-THC), which were negatively correlated with RFI, were related to the synthesis of primary bile acids (KEGG ID: M00120). C24:5n-6,9,12,15,18 (C24:5n-6) was involved in the metabolism of linoleic acid (KEGG ID: M00592). There are two metabolites belonging to the vitamin D category, including (10S)-1α,19,25-trihydroxy-10,19-dihydrovitamin D3 and (22E)-1α-hydroxy-22,23- didehydrovitamin D3. The above six metabolites in the negative ion mode were all negatively correlated with RFI. The metabolite 2-Keto-3-deoxy-D-gluconic acid (KDG) is involved in glucose metabolism (KEGG ID: M00052). The remaining two metabolites are 6-hydroxyhexanoic acid and m-coumaric acid.

### Lasso regression

**Fig. 4:**
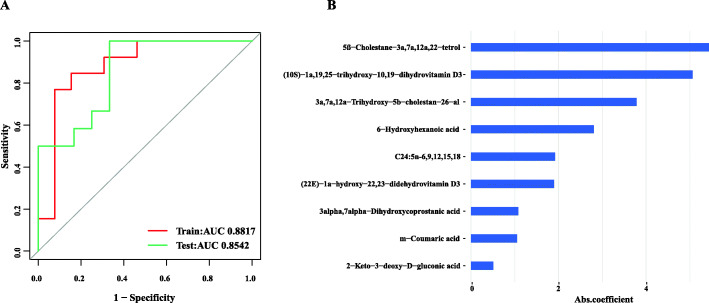
Assessing the weight of nine metabolites using Lasso regression analysis. **A** ROC curve of the training set (red) and the test set (green). **B** Regression coefficients of nine metabolites in the Lasso model. The y-axis of the graph on the right represents metabolites, and the x-axis represents the regression coefficient of metabolites

After identifying hub genes, we performed Lasso regression analysis on the nine annotated metabolites for feature selection. From the analysis results of WGCNA, we regrouped the samples into high- and low-FE groups based on the RFI values of individuals. To detect the quality of the model, the ROC curve was used to reflect its specificity and sensitivity. The results showed that the model has excellent prognostic effectiveness, with AUCs of the training set and the test set of 0.8817 and 0.8542, respectively (Fig. 4A). Subsequently, to evaluate the most valuable potential biomarkers, we ranked the importance of each metabolite according to the absolute value of the regression coefficient in the regression model (Fig. 4B).

## Discussion

Improving the feed efficiency traits of livestock is of great significance, but it is not easy to estimate. Therefore, any measure that can effectively predict feed efficiency is meaningful for production. Although there is currently much work to study FE at the genetic level, few studies have linked metabolites to feed efficiency phenotypic traits. In this study, we analyzed and compared the metabolites in the feces of pigs in the high-FE and low-FE groups by LC-MC technology and interpretation tools, including WGCNA and Lasso regression. To the best of our knowledge, this is the first report combining these methods to study the metabolomic profile related to feed efficiency and related traits in DLY pigs.

At present, FCR and RFI are commonly used to evaluate FE traits, and it is believed that RFI can better represent feed efficiency [2, 3, 14], which is consistent with our WGCNA analysis results. The RFI and FCR are continuously varying quantitative traits, and the factors affecting quantitative traits are diverse and have different weights. There are two strategies to analyze and study quantitative traits: (i) one is to group quantitative traits according to thresholds, our PCA and OPLS-DA analysis was to directly determine the experimental animals into two groups of high or low feed efficiency and then analyze them. This analysis method can identify the influencing factors that affect the phenotype with greater weight as soon as possible; (ii) another strategy is to correlate the values of quantitative traits directly with the influencing factors. The WGCNA correlation analysis we performed can more comprehensively take into account the continuity effect of metabolite changes on the phenotype. The two methods can play a complementary role, facilitating a more rapid and comprehensive search for factors influencing traits. In short, the two methods can play a complementary role, facilitating a quick and comprehensive search for factors affecting the trait.

In our data, we found that the use of powerful tools such as PCA and OPLS-DA were not sufficient to distinguish the different features between the high- and low-FE animals. There are many possible explanations for the unsatisfactory results of PCA and OPLS-DA, including but not limited to (1) the sampling process was carried out after the individual growth indicators were measured. When the pig reaches the weight (approximately 100 kg), its metabolic activity is often not as active as before, and the increase in weight has little effect on the growth performance of pigs after 100 kg [15]. Notably, collected fecal samples should be immediately stored at − 80 °C to − 20 °C temperature until processed to avoid microbial fermentation. Sample storage is a critical and sensitive step, and freeze-thaw cycles need to be minimized to prevent possible metabolite degradation [16]. Additionally, to maximize avoidance of additional variability, although difficult to achieve, we recommend collecting fecal samples from multiple time points per individual and analyze an aliquot of the homogenized and mixed samples, or by metabolic characterization of multiple samples from each animal to minimize this variability [17]; (2) throughout the experiment, all test subjects were clinically healthy. In contrast, liver metabolism and skeletal muscle metabolism are greatly affected in infected or inflamed piglets and a significant decrease in growth performance will be observed in growing pigs [18]. Therefore, there is no physiological interference between the FE groups that could cause large metabolome differences; (3) the number of animal individuals in our study (25 individuals per group) may not have high statistical power, so more animal groups and more targeted experimental designs may be needed to evaluate feed efficiency in the future. Because the results of the PCA and OPLS-DA models were not ideal, we then adopted WGCNA analysis to select the modules and metabolites most closely related to RFI and FCR. After screening and annotation, we obtained nine metabolites in these models. Based on these metabolites, we identified four pathways from the KEGG database that were also significantly related to feed efficiency, including lipid metabolism (primary bile acid synthesis, linoleic acid metabolism), vitamin D, and glucose metabolism. Moreover, the Lasso regression model showed that all nine annotated metabolites contribute to feed efficiency.

The metabolite 22-OH-THC is a kind of bile alcohol, which is the end product of catabolism of cholestanoic acids [19–21]. Bile alcohol may be regarded as an intermediate and side product from the normal pathways in bile acid biosynthesis [22]. Notably, THC26 and DHCA were mainly involved in the biosynthesis of primary bile acids. The specific synthesis process is that cholesterol 7α-hydroxylase (CYP27A1) catalyzes the oxidation of steroid side chains to form THC26 or DHCA in the mitochondria of liver cells and then obtains the primary bile acid cholic acid (CD) or chenodeoxycholic acid (CDCA) under the catalysis of various enzymes [23–27]. Interestingly, although the synthesis of bile acids is determined by a variety of cytochrome P450 enzymes (CYPs), both THC26 and DHCA are intermediate products catalyzed by CYP27A1 [28]. Bile acids start from the catabolism of cholesterol and are the final product of cholesterol catabolism; they play a critical role in food digestion and nutrient absorption, helping the absorption of lipids and fat-soluble vitamins in the intestine [27, 29–31]. After passing down the intestine with bile, approximately 95% of bile acids are reabsorbed in the terminal ileum and circulate back to the liver through the portal vein [23, 30, 32]. The performance of these functions of bile acid mainly depends on its enterohepatic circulation process, which is of great significance for nutrient absorption and distribution, metabolic regulation and homeostasis [23, 30, 32–34]. The results of metabolite network analysis showed that three metabolites related to bile acid synthesis were significantly negatively correlated with RFI traits, which means that they were positively correlated with feed efficiency. This result was consistent with our finding that these metabolites had higher levels in the high feed efficiency group. At present, little is known about the transport of bile acids and intermediates between different chambers, which may provide some references for understanding the important factors in the synthesis of bile acids.

In addition, (10S)-1α,19,25–trihydroxy-10,19-dihydrovitamin D3 and (22E)-1α-hydroxy–22,23-didehydrovitamin D3 belong to vitamin D, which is a steroid derivative [35, 36]. Vitamin D has various effects on lipid metabolism and immune system function through its effects on nuclear hormone receptors (such as vitamin D receptor and PPARγ) [37, 38]. Similarly, our results were consistent with the effect of vitamin D on lipid metabolism mediated through these receptors. Previous studies have shown that the CYP27A1 enzyme can catalyze the hydroxylation of compounds both in the biosynthesis of bile acids and the bioactivation of vitamin D3 [39–41]. The acidic pathway (or alternative pathway) of bile acid synthesis is initiated by CYP27A1, which is a mitochondrial cytochrome P450 enzyme widely distributed in most tissues and macrophages [23, 26]. CYP27A1 cannot only catalyze the 25-hydroxylation of vitamin D3, which is required for the conversion of vitamin D3 into a functionally active form, but may also regulate cholesterol homeostasis by promoting the synthesis of bile acids or producing active oxysterols [26, 41–43]. Although there are no current reports on the effect of adding this enzyme, this warrants further research. Additionally, metabolite C24:5n-6 was involved in the alpha linolenic acid and linoleic acid metabolism pathways [44]. Linoleic acid is the main dietary n-6 polyunsaturated fatty acid (PUFA), and livestock mainly obtain it from diets such as vegetable oil, soybeans, and corn [45]. Previous studies reported that higher n-6 PUFA intake can reduce liver fat in overweight individuals, improve liver metabolism, and regulate the balance between fatty acid oxidation and lipid synthesis [46, 47]. In the process of linoleic acid metabolism, linoleic acid is catalyzed by the rate-limiting enzyme fatty acid desaturase-2 (FADS2), and after a series of extensions, C24:5n-6 is produced by FADS2 catalyzed C24:4n-6 [48]. Our results showed that C24:5n-6 correlated negatively and significantly with RFI traits and was significantly higher in the high-FE group than in the low-FE group (*p* = 0.002). Notably, in the process of linoleic acid metabolism (elongation and desaturation), there was no significant difference between the upstream metabolite linoleic acid in the high and low FE groups, while the downstream C24:5n-6 was extremely different in the two groups. Besides, little research has been conducted on these three metabolites (m-coumaric acids, 6-hydroxyhexanoic acid and 2-keto-3-deoxy-d-gluconic acid), and there is still insufficient evidence to show that they are closely related to feed efficiency. We hope that with the continuous updating and refining of metabonomics database, we will have enough information to intensively elaborated them in the future. Based on this, these findings can also provide some references for further analysis of linoleic acid metabolism. Improving feed efficiency is a concern, but these enzyme and potential metabolic markers deserve further evaluation and research to clarify their biological significance.

## Conclusions

In this study, the PCA and OPLS-DA models have low explanatory variances for pig feed efficiency phenotypes. Then, through WGCNA coexpression analysis, we found two important modules and 210 potential metabolic features related to feed efficiency in the positive and negative modes. The nine potential metabolic markers obtained from screening and annotation were assessed by the Lasso model, which offered possibilities of collecting metabolic hallmarks in feces under minimizing stress reactions in the future. In short, this study identified important pathways and candidate biomarkers closely related to RFI, that contribute to understanding the molecular mechanism of feed efficiency more comprehensively and provides an important reference for further verifying the application of metabolite signatures to identify pig feed efficiency traits.

## Methods

### Animals and sample collection

A total of 225 female DLY pigs in this study were provided by Guangdong Wen’s Foodstuffs Group Co., Ltd. (Yunfu, China). The experimental animals were randomly divided into 30 pens, and each pen had 6 to 8 pigs. All pigs were arranged in a controlled environment with free access to water and food. The pig farm has strict epidemic prevention measures, the living conditions of the pigs such as humidity and temperature are controlled by sophisticated equipment, and veterinarians also regularly check the health status of the pigs to ensure that the pigs we randomly selected in the future are healthy throughout the entire cycle. Phenotypic data such as feed intake and weight per meal were recorded by the Osborne Feed Intake Recording Equipment (FIRE) Pig Performance Testing System (Osborne, KS, United States). The entire experimental process lasted approximately 12 weeks from an initial weight of approximately 30 kg to 100 kg. The calculation methods for FCR and RFI are based on previously described studies [49]. In this study, FE was defined as the weight gain from 30 kg to 100 kg of body weight divided by the total feed intake, which is the inverse of the commonly used feed conversion ratio (FCR). It means low FCR and RFI ratios correspond to high FE. After ranking the FCR values of 225 pigs, the top 25 highest FCR and 25 lowest FCR were selected as the Low-FE and High-FE groups, respectively. Fecal samples of 50 female piglet were collected after rectal stimulation. From each pig 2–3 tubes (2-ml sterile tubes) of fecal samples were collected, and the tubes were immediately transferred to liquid nitrogen for temporary storage. Then, to minimize the effects of microbial fermentation, the samples were stored at − 80 °C until analysis. In the entire monitored experiment, except for the sampling required by the research, no behaviors that caused animal stress reactions were carried out. The experimental protocol used in this study is in accordance with the Animal Protection and Use Committee of South China Agricultural University (SCAU, Guangzhou, China) (Approval number SCAU#0017).

### Fecal sample pretreatment

Fifty milligrams of fecal sample were accurately weighed, and 400 μl of extraction solution (methanol: water = 4:1) was added to the samples. Then, the high-throughput tissue grinder was used to crush at low temperature (60 Hz, − 20 °C). After vortex mixing and ultrasound at 40 kHz for 30 min at 5 °C, the extracted samples were placed at − 20 °C for 30 min. The solution was then centrifuged at 13,000 g for 15 min (4 °C), and the supernatant was extracted and injected into the LC-MS system for analysis.

### LC-MS analysis and quality control

The instrument platform for LC-MS analysis in this study was the AB SCIEX UPLC-TripleTOF system. The chromatographic conditions were as follows: the column was a BEH C18 column (100 mm × 2.1 mm i.d., 1.7 μm; Waters, Milford, USA); mobile phase A was water (containing 0.1% formic acid), and mobile phase B was acetonitrile/isopropanol (1/1) (containing 0.1% formic acid). The solvent gradient changed according to the following conditions: from 0 to 3 min, 95% (A): 5% (B) to 80% (A): 20% (B); from 3 to 9 min, 80% (A): 20% (B) to 5% (A): 95% (B); from 9 to 13 min, 5% (A): 95% (B) to 5% (A): 95% (B); from 13 to 13.1 min, 5% (A): 95% (B) to 95% (A): 5% (B), from 13.1 to 16 min, 95% (A): 5% (B) to 95% (A): 5% (B) for equilibrating the systems. The sample injection volume was 20 uL and the flow rate was set to 0.4 mL/min. The column temperature was maintained at 40oC. During the period of analysis, all these samples were stored at 4oC. The flow rate was 0.40 mL/min, the injection volume was 20 μL, and the column temperature was 40 °C. In addition, the sample mass spectrometer signals were collected using positive and negative ion scanning modes. The mass spectrometry conditions were as follows: electrospray capillary voltage, injection voltage and collision voltage: 1.0 kV, 40 V and 6 eV; ion source temperature and desolvation temperature: 120 °C and 500 °C; carrier gas flow rate: 900 L/h; mass spectrometry scan range: 50–1000 m/z; resolution: 30,000.

To evaluate the stability of the analysis system and find the variables with large variations in the analysis system during analysis, all test samples were mixed as quality control (QC) samples. In the process of instrument analysis, a QC sample was inserted every 8–10 samples.

### Data analysis

Before conducting statistical analysis, the raw data were imported into the metabolomics software ProgenesisQI (Waters Corporation, Milford, USA) to generate the matrix of retention time, mass-to-charge ratio, and peak intensity for baseline filtering, peak identification, integration, retention time correction, and peak alignment. Furthermore, to obtain the final data matrix for subsequent analysis, the preprocessing process was as follows: (i) only variables with nonzero values above 80% in all samples were retained; (ii) missing values were filled using the k-nearest neighbors (KNN) approach in the R DMwR package; (iii) standardized values were obtained via the Z-score method, and the variables with relative standard deviation (RSD) ≥ 30% of the QC samples were deleted.

The *p* values for statistical differences in these phenotypes were based on ANOVA, Wilcoxon rank-sum test or unpaired Student t-tests, depending on the distribution of the data. If the data were normally distributed and homogenous, ANOVA was used to evaluate whether these traits were statistically significant, if the data were normally distributed but not homogeneous, unpaired Student t-tests were used, while a Wilcoxon rank-sum test was used otherwise. Principal component analysis (PCA) and (Orthogonal) partial least squares discrimination analysis (OPLS-DA) models were built using the ropls package in R [50]. PCA was used to observe the overall distribution between samples and the dispersion degree between groups. OPLS-DA was used to distinguish the different metabolites between groups. The goodness of fit (R^2^) and goodness of prediction (Q^2^) in cross-validation were used to evaluate the performance of the OPLS-DA model, and 500 permutation tests were performed.

### Weighted gene correlation network analysis

Network and clustering analyses were performed using the R package Weighted Gene Coexpression Network Analysis (WGCNA) [51]. The Pearson correlation coefficient was calculated to obtain a coexpression similarity measure and used to subsequently construct an adjacency matrix using soft threshold combined with topological overlap matrix (TOM). Then, hierarchical clustering was performed based on the TOM. Briefly, the soft thresholds of the positive and negative ion modes were set to 3 and 8, respectively, to achieve the approximate scale-free topology of the signed network (R^2^ > 0.9) (Fig. S3). In the dynamic tree cutting algorithm, deepSplit was set to 2 and minModuleSize was set to 50. The first principal component of the metabolite module was used as the feature vector of the module (including most of the variation information of all metabolites in the module), used to calculate the correlation coefficient between the metabolite module and feed efficiency, and then the most relevant module for subsequent analysis was selected. Subsequently, the gene significance (GS) and module membership (MM) of the most relevant module were calculated. Among these, GS can represent the correlation between metabolic characteristics and phenotype, and MM can represent the correlation between metabolic characteristics and module feature vectors. GS > 0.2 and MM > 0.8 were set as the threshold to screen the hub genes. Since WGCNA was first used for transcriptome data, we followed the term hub gene to represent the important metabolites identified. Subsequently, hub genes were identified by using the online Human Metabolome Database (HMDB) [52] and the METLIN public database [53]. The *p*-values of the hub genes were computed using the Wilcoxon test. The pathways in which hub genes participated were identified in the Kyoto Encyclopedia of Genes and Genomes (KEGG) database [54].

### Lasso-penalized linear regression

We performed the Lasso regression in R using the glmnet [55] and caret packages. The sample data were randomly divided into a training set and a test set at a 1:1 ratio. Ten cross-validations were performed to calculate the lambda value (lambda = 0.08678594). Receiver operating characteristic (ROC) curves were generated using the pROC curve, predictions were made on the training set and the test set, and the importance of the variables was evaluated by the varimp function of the caret package.

## Supplementary Information

**Additional file 1: Table S1**. The detailed information of experimental animals.

**Additional file 2:** **Table S2**. The nine metabolites in the positive and negative modes obtained by WGCNA.

**Additional file 3: Figure S1.** Principal Component Analysis (PCA) scores plots. The analysis was based on LC/MS data of fecal samples from H-FE (green) and L-FE (red) of (A) positive and (B) negative model. **Figure S2.** Clustering dendrogram and module-trait correlation plots. Each coloured row represents a colour-coded module which contains a group of highly connected metabolic features. A total of 14 and 14 modules was identified in (A) positive and (B) negative model, respectively. **Figure S3.** Soft-thresholding values estimation. Scale independence and mean connectivity of various soft-thresholding values (β) in (A) negative and (B) positive model.

## Data Availability

All data generated or analysed during this study are included in this published article and its supplementary files.

## Abbreviations

ADFI: Average daily feed intake
BW: Body weight
C24:5n-6: C24:5n-6,9,12,15,18
CA: Cholic acid
CDCA: Chenodeoxycholic acid
CYP27A1: Cholesterol 7α-hydroxylase
DHCA: 3alpha,7alpha-Dihydroxycoprostanic acid
FADS2: Fatty acid desaturase-2
FCR: Feed conversion ratio
FE: Feed efficiency
GS: Gene significance
H-FE: High feed efficiency
KDG: 2-Keto-3-deoxy-D-gluconic acid
L-FE: Low feed efficiency
MM: Module membership
OPLS-DA: Orthogonal partial least squares discriminant analysis
PCA: Principal component analysis
PUFA: Polyunsaturated fatty acid
RFI: Residual feed intake
THC26: 3a,7a,12a-Trihydroxy-5b-cholestan-26-al
WGCNA: Weighted gene co-expression network analysis
22-OH-THC: 5β-Cholestane-3α,7α,12α,22-tetrol
