# Supplementary material for: Using nontargeted LC-MS metabolomics to identify the Association of Biomarkers in pig feces with feed efficiency
Source: Porcine Health Manag. 2021 Jun 2;7:39. doi: 10.1186/s40813-021-00219-w (PMC8170940; doi:10.1186/s40813-021-00219-w)
Supplement: Supplementary file 3 — Additional file 3: Figure S1. Principal Component Analysis (PCA) scores plots. The analysis was based on LC/MS data of fecal samples from H-FE (green) and L-FE (red) of (A) positive and (B) negative model. Figure S2. Clustering dendrogram and module-trait correlation plots. Each coloured row represents a colour-coded module which contains a group of highly connected metabolic features. A total of 14 and 14 modules was identified in (A) positive and (B) negative model, respectively. Figure S3. Soft-thresholding values estimation. Scale independence and mean connectivity of various soft-thresholding values (β) in (A) negative and (B) positive model. [file 40813_2021_219_MOESM3_ESM.docx]

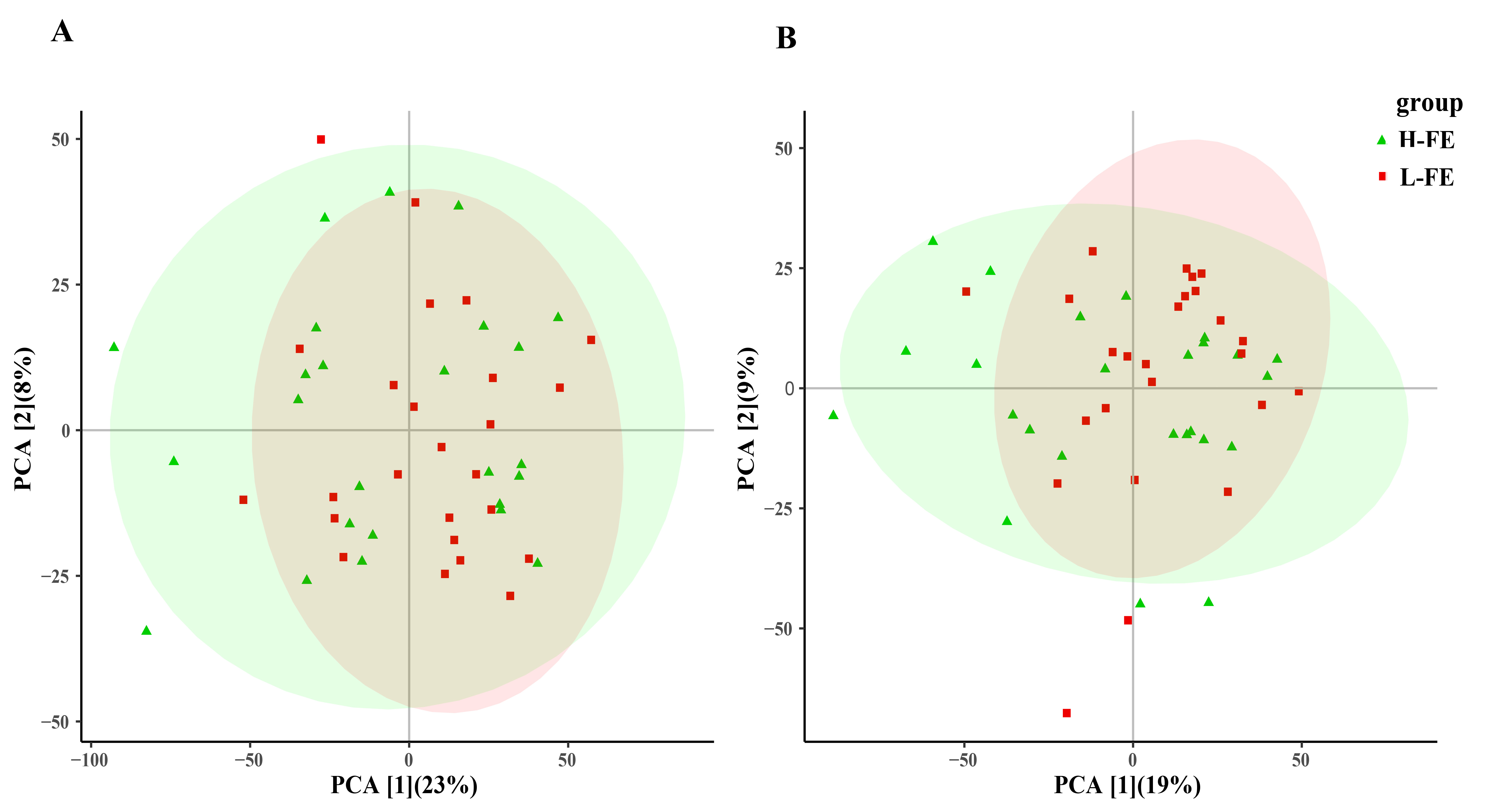


**Figure S1.** Principal Component Analysis (PCA) scores plots. The analysis was based on LC/MS data of fecal samples from H-FE (green) and L-FE (red) of (A) positive and (B) negative model.


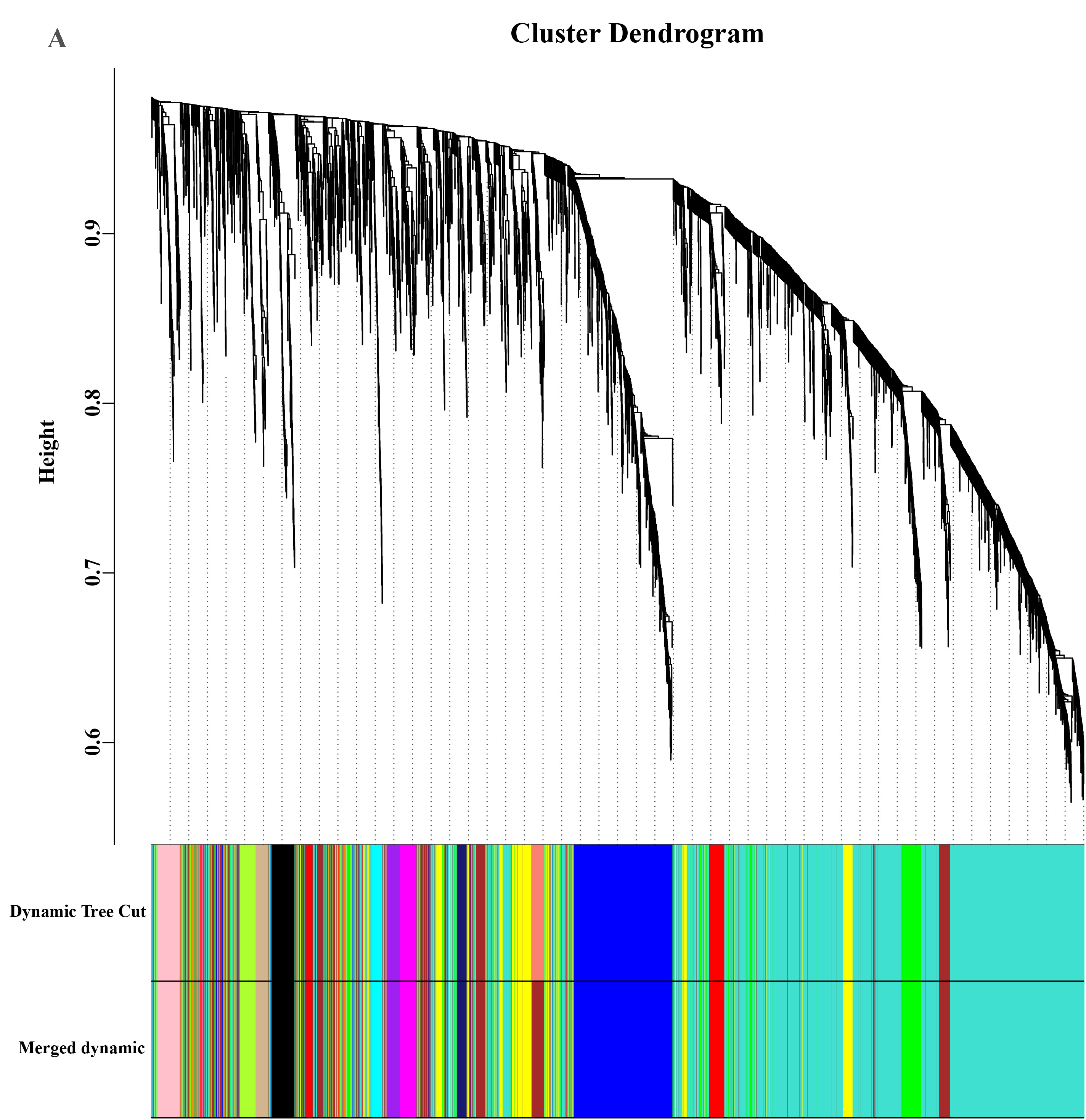


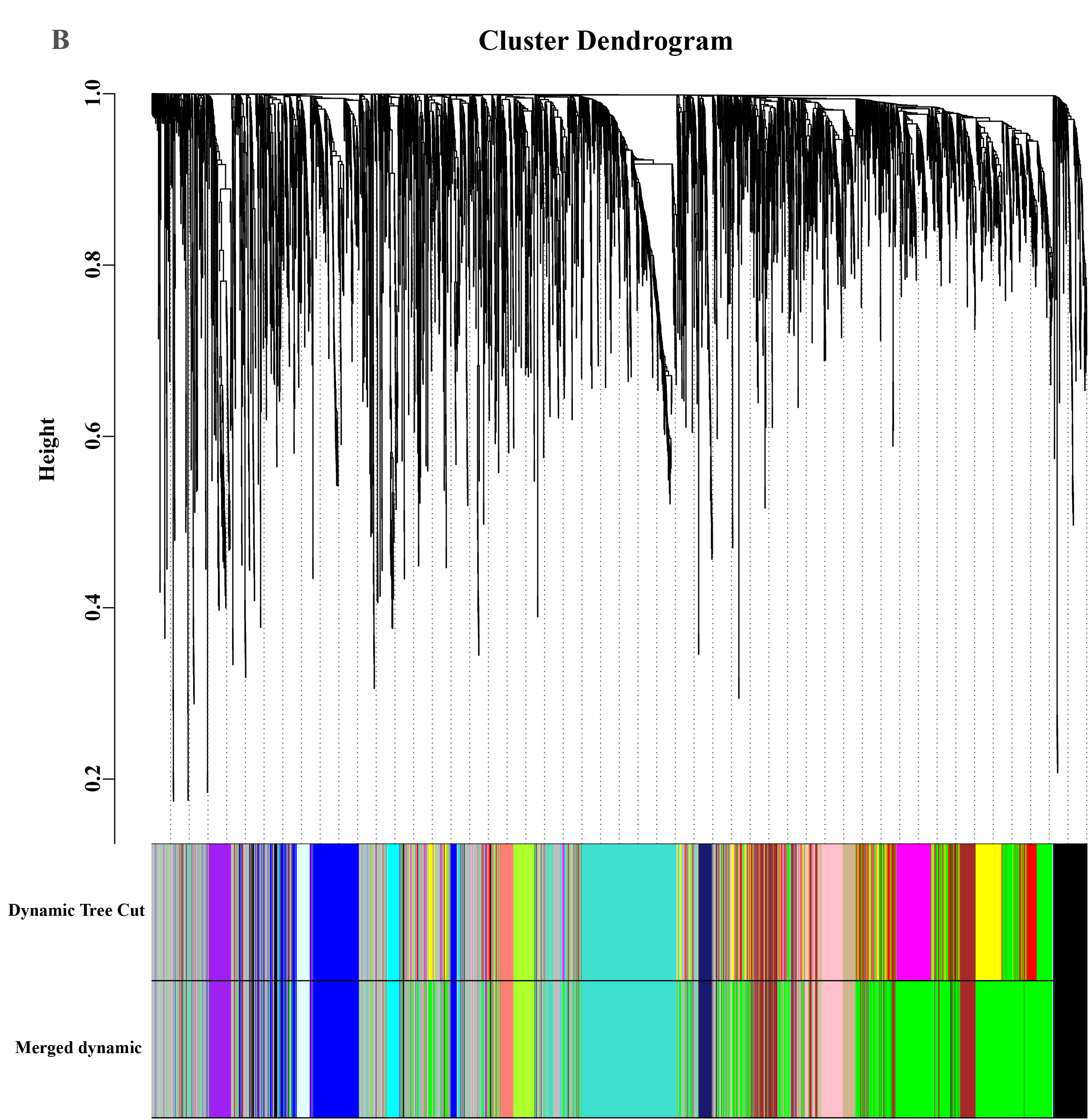


**Figure S2.** Clustering dendrogram and module-trait correlation plots. Each coloured row represents a colour-coded module which contains a group of highly connected metabolic features. A total of 14 and 14 modules was identified in (A) positive and (B) negative model, respectively.


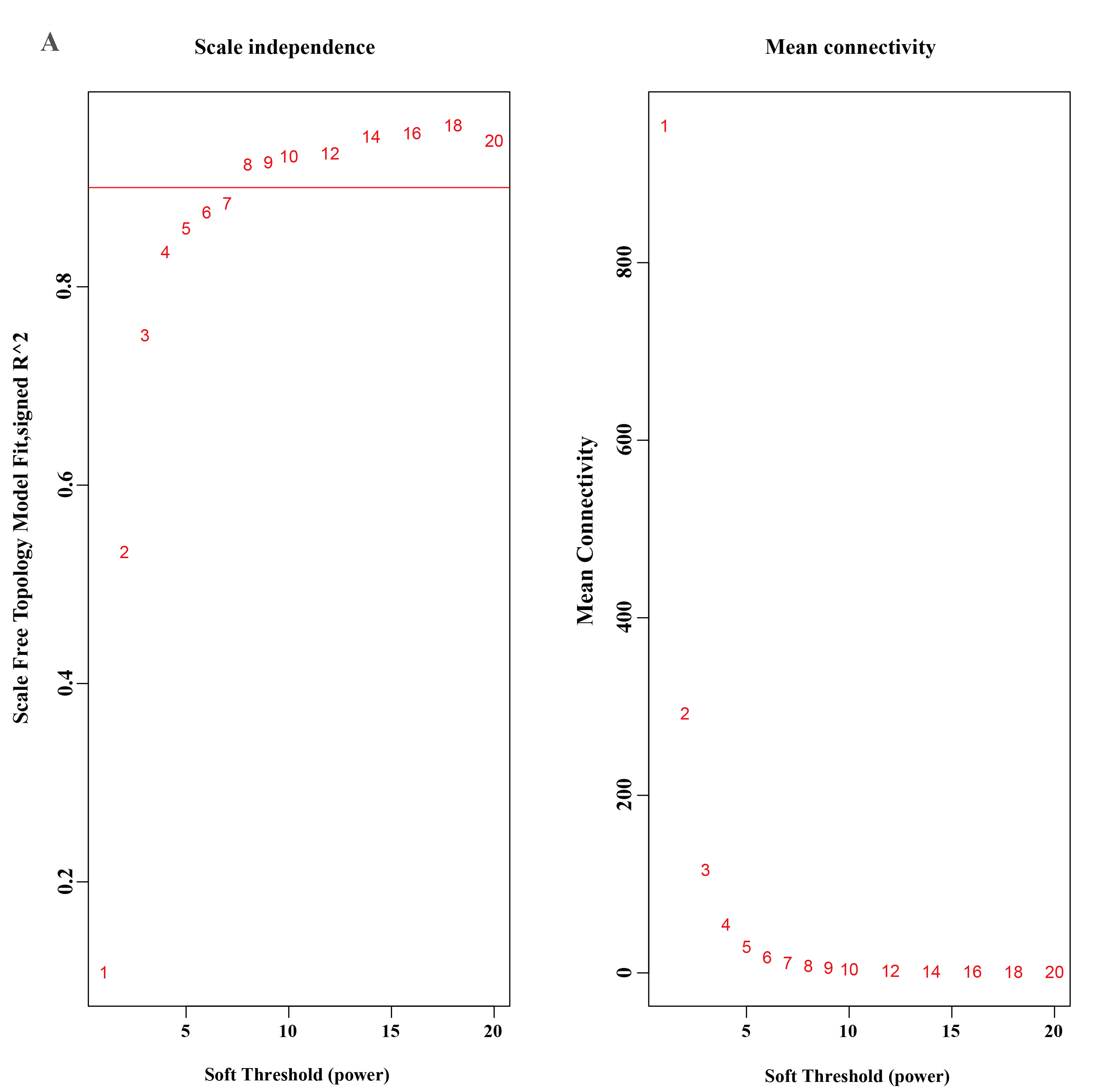


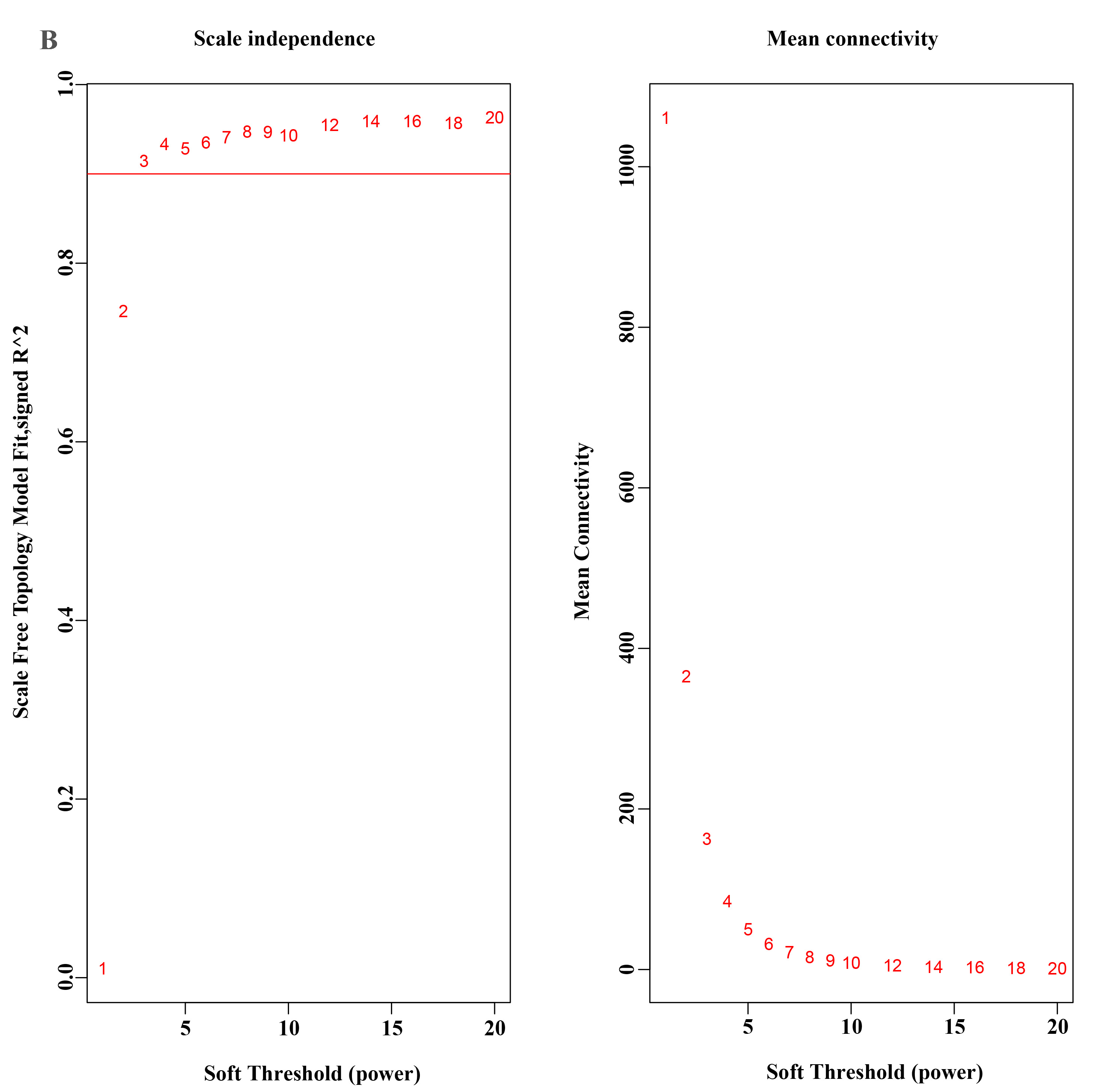


**Figure S3.** soft-thresholding values estimation. Scale independence and mean connectivity of various soft-thresholding values (β) in (A) negative and (B) positive model.
